# Immunoglobulin-Induced Aseptic Meningitis in Juvenile Dermatomyositis: A Case Report

**DOI:** 10.7759/cureus.31808

**Published:** 2022-11-22

**Authors:** Oi Man Chan, Chon In Kuok, Kwai Yu Winnie Chan, Hoi Man Roanna Yeung

**Affiliations:** 1 Paediatrics, The Chinese University of Hong Kong, Hong Kong, HKG; 2 Paediatrics, Queen Elizabeth Hospital, Hong Kong, HKG

**Keywords:** pediatric rheumatology, young child, juvenile dermatomysitis, drug-induced aseptic meningitis, intravenous immunoglobulin (ivig)

## Abstract

Aseptic meningitis is a known but unusual serious adverse effect of intravenous immunoglobulin (IVIG). It usually resembles infectious meningitis, which makes its diagnosis challenging. In this report, we present the case of a five-and-a-half-year-old Chinese girl with juvenile dermatomyositis (JDM) who presented with signs of meningismus 21 hours after the initiation of IVIG infusion. Her blood work at diagnosis showed neutrophilia and lymphopenia. The cerebrospinal fluid (CSF) analysis demonstrated neutrophilic pleocytosis, hyperproteinorrachia, and normoglycorrhachia. All microbiological tests were negative. The child fully recovered within 72 hours without neurological sequelae. IVIG-induced aseptic meningitis remains a diagnosis of exclusion. Although it is rare, pediatricians should be aware of this complication and avoid unnecessary investigations or treatment.

## Introduction

Aseptic meningitis is a known severe adverse effect of immunoglobulin. It is rare and usually resembles infectious meningitis, which makes its diagnosis challenging. Its causes are either infectious or non-infectious (also called aseptic), consisting of autoimmune, neoplasia, and iatrogenic etiologies [1].

Intravenous immunoglobulin (IVIG) is widely used for a broad range of diseases, including immunodeficiencies, neuromuscular diseases, and autoimmune diseases. The treatment of juvenile dermatomyositis (JDM) with IVIG is generally considered effective as a second-line option, particularly for steroid-resistant patients or those with predominant skin activity [2-4]. A retrospective JDM cohort in 2011 demonstrated IVIG efficacy in controlling disease activity [5]. We discuss the case of a five-and-a-half-year-old girl with JDM, who experienced no adverse events with initial IVIG (Intragam P) administration, but subsequently developed aseptic meningitis after switching to another formulary IVIG (Privigen). The possible risk factors for developing aseptic meningitis are also discussed.

This article was previously posted to the ResearchSquare preprint server in November 2021.

## Case presentation

**Figure 1:**
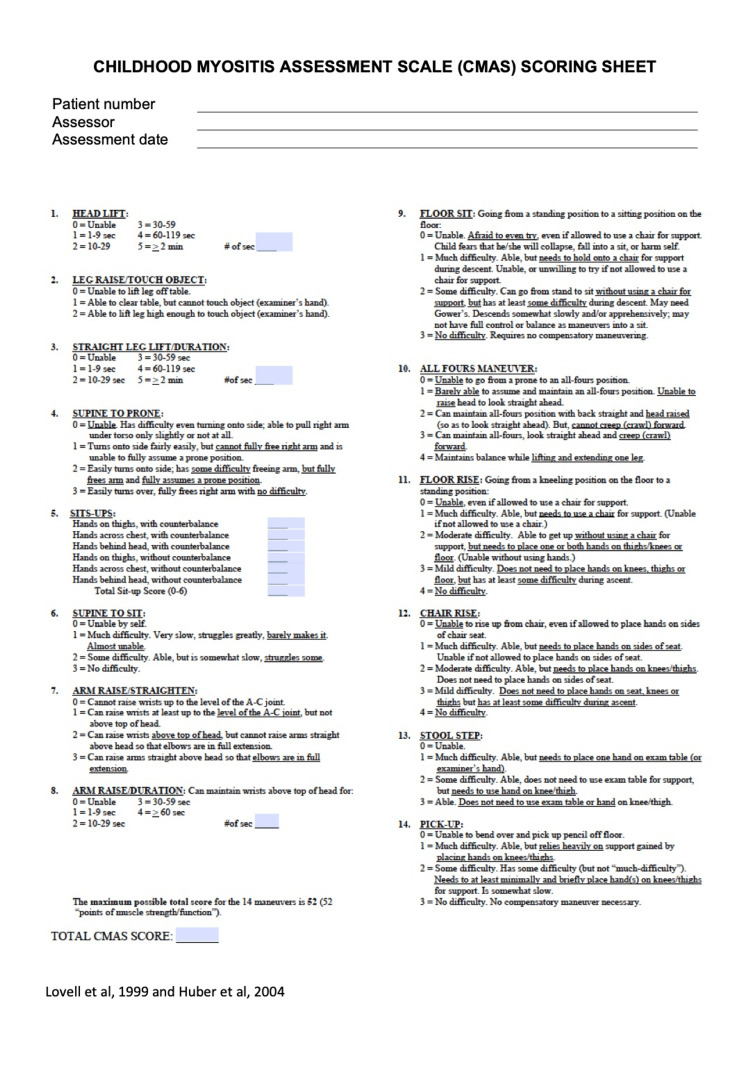
Childhood Myositis Assessment Scale (CMAS) scoring sheet 1. Lovell DJ, Lindsley CB, Rennebohm RM, et al.: Development of validated disease activity and damage indices for the juvenile idiopathic inflammatory myopathies. II. The Childhood Myositis Assessment Scale (CMAS): a quantitative tool for the evaluation of muscle function. The Juvenile Dermatomyositis Disease Activity Collaborative Study Group. Arthritis Rheum. 1999, 42:2213-2219. 10.1002/1529-0131(199910)42:10<2213::Aid-anr25>3.0.Co;2-8 2. Huber AM, Feldman BM, Rennebohm RM, et al.: Validation and clinical significance of the Childhood Myositis Assessment Scale for assessment of muscle function in the juvenile idiopathic inflammatory myopathies. Arthritis Rheum. 2004, 50:1595-1603. 10.1002/art.20179

Our patient was a Chinese girl who initially presented with proximal muscle weakness and limping gait at four years of age. She also demonstrated dermatological features including heliotrope rash over the facial region and Gottron papules over her metacarpophalangeal and interphalangeal joints with cutaneous ulcerations. She had raised serum creatine kinase up to 3900 IU/L, and her MRI showed features of myositis over her bilateral shoulders, arms, and thigh muscles. She was given the diagnosis of JDM. Her Childhood Myositis Assessment Scale (CMAS) score (see the CMAS score sheet, Figure 1, in appendices) was 5/52 points (Table 1).

**Table 1:** Childhood Myositis Assessment Scale (CMAS) score

Maneuver	Patient score
1. Head elevation (neck flexion)	1: 1–9 seconds
2. Leg raise/touch object	0: unable to lift the leg off the table
3. Straight leg lift/duration	0: unable
4. Supine to prone	1: turns onto the side fairly easily, but cannot fully free arms and is not able to fully assume a prone position
5. Sit-ups	0: unable
6. Supine to sit	0: unable
7. Arm raise/straighten	1: can raise wrists at least up to the level of the acromioclavicular joint but not above the top of the head
8. Arm raise/duration	1: 1–9 seconds
9. Floor sit	0: unable, afraid to even try
10. All-fours maneuver	0: unable to go from a prone to an all-fours position
11. Floor rise	0: unable, even if allowed to use a chair for support
12. Chair rises	0: unable to rise from the chair, even if allowed to place hands on sides of the chair
13. Stool step	1: much difficulty. Able, but needs to place one hand on the exam table
14. Pick-up	0: unable to bend over and pick up a pencil off the floor

The patient was initially treated with intravenous methylprednisolone, subcutaneous methotrexate, and six doses of monthly IVIG (Intragam P) at presentation. The IVIG was administered at a dosage of 1 gram/kg (total of 15 grams in the volume of 250 ml) over nine hours each day for two consecutive days. She had no adverse reaction to the medication. She responded well to treatment and her muscle power improved with a CMAS of 43 out of 52 four months later. Her steroid was gradually tapered down, but she developed worsening vasculitis with necrotic lesions and chondritis over both ears. Her skin condition remained refractory despite increasing the steroid and methotrexate dosage, as well as adding oral cyclosporin A. Cyclosporin A was subsequently switched to oral cyclophosphamide. Two days after the switch, IVIG was given additionally to optimize the control of her skin activity at the age of five-and-a-half years. Due to a change in our hospital drug formulary, the patient received Privigen instead. She was given 2 grams/kg (total of 32.5 grams in the volume of 325 ml), which was infused over 11 hours.

She was asymptomatic during the infusion but developed a high fever with a temperature of 40 ºC, headache, and repeated vomiting 10 hours after the completion of the injection. She also complained of neck pain and photophobia. There was no seizure, altered state of consciousness, or abnormal behavior. Physical examination demonstrated meningismus with neck stiffness and positive Kernig sign. There was no other focal neurological deficit.

Her blood tests (Table 2) showed an elevated white cell count at 24 x 10^9^/L with neutrophilia (absolute neutrophil count: 17.3 x 10^9^/L), but normal C-reactive protein (<0.7 mg/L) and serum procalcitonin level (0.14 ng/ml). The CT of the brain was normal. The cerebrospinal fluid (CSF) was clear and colorless. Its analysis demonstrated neutrophilic pleocytosis with 2188 cells/mm^3^ (87% neutrophils) without eosinophils, hyperproteinorrachia (0.66 g/L), and normoglycorrhachia (2.9 mmol/L in CSF and 4.7 mmol/L in plasma). She was initially treated with an intravenous meningitic dose of meropenem and acyclovir. Meropenem was chosen because of the abundance of extended-spectrum beta-lactamase-producing Enterobacteriaceae (ESBL-E) in the Hong Kong community [6]. The cultures from her blood sample and CSF did not isolate any causative organisms.

**Table 2:** Investigation results CSF: cerebrospinal fluid; PCR: polymerase chain reaction; DNA: deoxyribonucleic acid; RNA: ribonucleic acid

Laboratory tests	Patient values	Reference values
Blood tests		
White cell count	24 x 10^9^/L	5.0-15.0 x 10^9^/L
Neutrophil count	17.3 x 10^9^/L	1.5–8.0 x 10^9^/L
C-reactive protein	<0.7 mg/L	<5.0 mg/L
Procalcitonin	0.14 ng/mL	<0.5 ng/mL
The blood culture showed no growth after 5 days of incubation		
First CSF results		
White cell count	2188/cubic mm	
Neutrophils	87%	
Protein	0.66 g/L	0.15–0.45 g/L
Glucose	2.9 mmol/L	2.2–3.9 mmol/L
Plasma glucose	4.7 mmol/L	2.5–11.0 mmol/L
CSF PCRs of the following were negative: herpes simplex virus type 1 and type 2 DNA, varicella-zoster virus DNA and enterovirus RNA, Escherichia coli K1 DNA, Haemophilus influenzae DNA, Listeria monocytogenes DNA, Neisseria meningitidis (encapsulated) DNA, Streptococcus agalactiae DNA, Streptococcus pneumoniae DNA, cytomegalovirus DNA, human herpesvirus 6 DNA, Cryptococcus neoformans/gattii DNA		
CSF showed a negative gram stain and the culture showed no growth after 7 days of incubation		
CSF fungal culture showed no fungus isolated after 14 days of incubation		
Second CSF results		
White cell count	11/cubic mm	
Neutrophils	9%	
Protein	0.19 g/L	0.15–0.45 g/L
Glucose	3.6 mmol/L	2.2–3.9 mmol/L
Plasma glucose	5.3 mmol/L	2.5–11.0 mmol/L

A lumbar puncture was repeated on day six and CSF analysis revealed a white cell count of 11 cells/mm^3^ with 9% polymorphs, normal protein (0.19 g/L), and normal glucose levels (3.6 mmol/L in CSF and 5.3 mmol/L in plasma) (Table 2). The patient completed a 14-day course of meropenem. Based on the temporal relation between IVIG administration and the symptoms onset, sterile cultures, as well as the normal C-reactive protein and procalcitonin levels, a diagnosis of aseptic meningitis was made.

Her symptoms and fever subsided on day two of admission, and she recovered completely without any neurological sequelae.

## Discussion

IVIG-induced aseptic meningitis is a rare complication, with an estimated incidence of 0.6-1% [7]. Apart from being used in the treatment of immune thrombocytopenia (ITP), IVIGs are also being given to patients as a treatment for various autoimmune and inflammatory diseases such as Kawasaki disease, Guillain-Barre syndrome, and chronic inflammatory demyelinating polyneuropathy. The exact pathophysiology of IVIG-induced aseptic meningitis remains unclear, with postulated mechanisms including direct toxic effect and immunological hypersensitive reaction [8].

Our patient did have the typical features of IVIG-associated aseptic meningitis described in the literature (Table 3) [7,9]. Firstly, there was a temporal relationship between the onset of the symptoms of aseptic meningitis and high-dose IVIG therapy. Secondly, the symptoms and signs of meningismus quickly resolved within 48 hours in our patient. Thirdly, although the initial bloodwork revealed leukocytosis and neutrophilia, and the CSF analysis showed neutrophilic pleocytosis, all cultures were negative. All these fit into the picture of aseptic meningitis induced by IVIG as described by Bharath et al. and Kemmotsu et al.

**Table 3:** Typical symptoms and signs of IVIG-induced aseptic meningitis IVIG: intravenous immunoglobulin; CSF: cerebrospinal fluid

	Bacterial meningitis	IVIG-induced aseptic meningitis
Symptoms	Headache, vomiting, photophobia, and fever	
Signs	Nuchal rigidity, positive Kernig sign without focal neurological sign	
CSF Leukocytes/mm^3^	500–10,000	100–2000
Main leukocyte type	Neutrophils	
Other criteria	Bacteria in CSF	Sterile CSF; temporal relationship with IVIG infusion
Management	Antibiotics	Drug discontinuation; symptomatic treatment
Time to regression	7–21 days	Within 2–3 days

Evidence concerning both patient and IVIG-related risk factors remains controversial. It has been suggested that a history of migraines, female sex, and underlying connective tissue disease such as systemic lupus erythematosus could be potential risk factors for developing aseptic meningitis following IVIG administration [10-12]. Further evidence is needed to evaluate whether JDM could also be a risk factor.

It is important to note that this was not the first time our patient had received IVIG. She developed aseptic meningitis after IVIG therapy was given at a higher dose (2 grams/kg) and faster rate (over 11 hours). Besides, our patient received a different brand of IVIG (Privigen) from her previous IVIG infusions (Intragam P) prior to the incidence of aseptic meningitis. Regarding IVIG-related risk factors, fast infusion rate and high initial dosage of IVIGs are thought to be risk factors for developing aseptic meningitis, but it is not always the case (Table 4). There is no concrete evidence as to whether different IVIG brands have various potentials in causing aseptic meningitis. It was thought that IgA concentration might be related. The administration of IVIG containing IgA may cause dramatic clinical reactions in patients with serum anti-IgA [1,13]. The IgA contents in both Privigen and Intragam were not specified, with Privigen stating a maximum of 0.025 mg/ml and Intragam P reporting <0.025 mg/ml (Table 5). Although 50% of patients developed aseptic meningitis after Privigen infusion in Bharath et al.'s retrospective study, due to the small number of patients, the brands of IVIG or varying commercial preparations have not been identified as risk factors.

**Table 4:** Documented cases of IVIG-related aseptic meningitis IVIG: intravenous immunoglobulin; WBC: white blood cell; CSF: cerebrospinal fluid; PEG: polyethylene glycol

	Source	Number of patients	Diagnosis	Age (years)	IVIG dose	IVIG duration (hours)	Brand	Onset	WBC x 10^9^/L CSF	CSF cytosis	Treatment	Time to recovery
1	Rao et al. [14]	1	Idiopathic thrombocytopenic purpura	9	0.4 g/kg	4	Sandoglobulin	12 hours after the last dose	2.5	98%	Prednisone 3 mg/kg for 4 days	48 hours
2	Mitterer et al. [15]	1	Idiopathic thrombocytopenic purpura	4	0.4 g/kg	Non-specified	Sandoglobulin	Second day	2	67%	Self-limiting	2 days
3	Chaabane et al. [16]	1	Idiopathic thrombocytopenic purpura	14	0.4 g/kg	Non-specified	Non-specified	2 days after the last infusion	0.14	70%	Floctafenine	3 days
4	Casteel-Van et al. [17]	1	Idiopathic thrombocytopenic purpura	7	0.4 g/kg	11	Sandoglobulin	Third day	Non-specified	Non-specified	Non-specified	Non-specified
5	Sekul et al. [18]	1	Dystrophy	7	2 g/kg	Non-specified	Non-specified	Within 24 hours	0.22	85%	Narcotic analgesics and antiemetic agents	3–5 days
6	Kato et al. [19]	1	Idiopathic thrombocytopenic purpura	2	0.4 g/kg/day for 5 days	Non-specified	Prepared with PEG	7 days after therapy	0.451	9%	Self-limiting	1 day
7	Casteels-Van et al. [20]	1	Idiopathic thrombocytopenic purpura	7	0.4 g/kg for 5 days	11	Sandoglobulin	1 hour after the completion of the second dose	2.45	88%	Self-limiting	1 day
8	Pallares and Marshall [21]	1	Idiopathic thrombocytopenic purpura	10	1 g/kg	6	Sandoglobulin	At the beginning of the second dose	2.86	97%	Cefotaxime for 72 hours	2 days
9	Obando et al. [22]	2	Idiopathic thrombocytopenic purpura	6–10	1 g/kg/day for 2 days	8–12	Flebogamma	10-12 hours after the secondinfusion	0.65–7.44	60–98%	Cefotaxime for <48 hours, analgesics	24–48 hours
10	Boyce and Spearman [23]	1	Kawasaki syndrome	9	2 g/kg	14	Polygam	10 hours after the last infusion	1.515	99%	Ceftriaxone for 72 hours	5 days
11	Preinger-Shapiro et al. [24]	1	Acquired immune neutropenia	2	1 g/kg/day for 4 days	Non-specified	Sandolglobulin	During the second infusion	3.5	95%	Self-limiting	24 hours
12	Kressebuch et al. [25]	2	Idiopathic thrombocytopenic purpura	7–8	0.4 g/kg/day	Non-specified	Sandoglobulin	12 hours after the second infusion; during the third infusion	0.667–1.62	92–95%	Self-limiting	Within a few hours
13	Kemmotsu et al. [9]	4	Kawasaki disease	1–10	1-2 g/kg	Non-specified	Sulfonated/Peg-treated	Within 25–40 hours from the start of the infusion	0.021–1.248	13–89%	Methylprednisolone 15 mg/kg in 2 children, self-limiting in 2 children	1–2 days
14	Kaarthigeyan and Burli [26]	1	Common variable immunodeficiency	10	0.4 g/kg	Non-specified	Non-specified	10 days after the last infusion	0.225	Non-specified	Ticarcillinclavulanate and ofloxacin	Within 1 week
15	Jain et al. [27]	1	Guillain-Barre syndrome	14	0.4 g/kg/day for 5 days	Non-specified	Non-specified	Fourth day	0.0000018	15%	Hydration, analgesics	2 days
16	Vassalini et al. [28]	1	Acute Epstein-Barr virus infection	4	0.4 g/kg	Non-specified	Non-specified	6 hours after the second infusion	2.993	84%	Ceftriaxone, dexamethasone	36 hours
17	Kattamis et al. [29]	2	Idiopathic thrombocytopenic purpura	8–11	1 g/kg/day for 2 days	Non-specified	Venoglobulin	Non-specified	0.635–2.237	64–91%	Non-specified	Non-specified

**Table 5:** Compositions of Privigen and Intragam P IgG: immunoglobulin G; IgA: immunoglobulin A

	Privigen	Intragam P
IgG1	67.8%	61%
lgG2	28.7%	36%
lgG3	2.3%	3%
lgG4	1.2%	1%
lgA	Maximum of 0.025 mg/ml	<0.025 mg/ml
Excipients	L-Proline, WFI	Maltose 10 mg/ml

Supportive measures such as analgesics and antiemetics seem to be sufficient in these cases [30]. Corticosteroids do not seem to be effective in treating IVIG-induced aseptic meningitis [9,10,31]. Stiehm suggested that steroids or anti-TNF could be used in severe cases of aseptic meningitis [32]. However, in Kemmotsu et al.'s study, there were no differences in the clinical courses between patients who received no medical treatment and those treated with intravenous methylprednisolone [9]. Re-infusions are not contraindicated [7,27]. In case our patient requires IVIG in the future, Privigen will be avoided. Switching to subcutaneous preparation could potentially be an effective strategy in attenuating adverse effects [33]. Subcutaneous immunoglobulin (SCIG) was associated with lower rates of aseptic meningitis [10]. Several studies have shown that SCIG can be used in treating various diseases including immunodeficiency diseases, myasthenia gravis, chronic inflammatory demyelinating polyneuropathy, etc. Further research is needed to determine its efficiency as an immunomodulatory therapy. Preventive measures including infusing at a slow rate, pre-hydration, and adequate fluid intake throughout infusion, as well as premedication with acetaminophen and antihistamine, could be considered [31].

Milder cases of aseptic meningitis might not necessarily be recognized, given that aseptic meningitis is such a rare complication of IVIG. On the other hand, post-IVIG headache is common. The true incidence of IVIG-induced aseptic meningitis could be under-reported. Although there is increasing evidence on the self-limitedness of IVIG-induced aseptic meningitis and its temporal profile, the necessity for lumbar puncture and antibiotics remains controversial [7,34]. Given that our patient had been treated with several immunomodulatory medications and her increased risks for opportunistic infections, she was treated with intravenous antibiotics.

## Conclusions

IVIG-induced aseptic meningitis remains a diagnosis of exclusion. Although it is rare, pediatricians should be aware of this complication and avoid unnecessary investigations or treatment. Supportive measures seem to be sufficient in these patients. Children usually recover without neurological sequelae.
